# Editorial: Case Reports in Pediatric Rheumatology 2022

**DOI:** 10.3389/fped.2023.1137843

**Published:** 2023-02-06

**Authors:** Maryam Bakhtiari Koohsorkhi, Junfeng Wu, Vahid Ziaee

**Affiliations:** ^1^ Children's Medical Center, Pediatrics Center of Excellence, Tehran, Iran; ^2^ Department of Rheumatology and Immunology, Children's Hospital of Chongqing Medical University, Chongqing, China; ^3^ Department of Pediatrics, Tehran University of Medical Sciences, Tehran, Iran; ^4^ Pediatric Rheumatology Research Group, Rheumatology Research Center, Tehran University of Medical Science, Tehran, Iran; ^5^ Pediatric Rheumatology Society of Iran, Tehran, Iran

**Keywords:** monogenic lupus, a20 haploinsufficiency, kabuki syndrome, factor h deficiency, bardet-Biedl syndrome, lupus podocytopathy, knee lipoma, MIS-C

**Editorial on the Research Topic** Case Reports in Pediatric Rheumatology 2022

The field of pediatric rheumatology is thriving and growing in science. the body of clinical knowledge and scientific work in this area has expanded exponentially and is receiving worldwide attention. the recently discovered field of autoinflammatory diseases describes disorders that are at the crossroads of immunology and rheumatology and the joint effort is shedding new light on the unclear pathogenesis, and helping in the identification of new treatment options. Systemic autoinflammatory diseases (SAIDs) consist of multisystem immune dysregulation disorders caused by the dysfunction of the innate immune system in the absence of infections or autoimmunity (1, 2).

For nearly 3 years, the main focus of the academic world has mostly been on COVID-19, presentations, complications, and treatment strategies. however, it seems like the appropriate time to attract attention and put emphasis on case reports, unusual presentations, and new and experimental treatment options for other rheumatologic diseases. Case reports count as a great source of new ideas and information in clinical medicine and it has the ability to report original discoveries and novel treatment strategies.

The present Special Issue entitled **“A Review on new Case Reports in Pediatric Rheumatology 2022”** aims to explore the advances in pediatric rheumatology and case reports chosen in this research topic represent some of the fresh advances in the field.

The recent discovery of monogenic inborn errors of immunity and the so-called subgroup of autoinflammatory disorders has broadened the field of pediatric rheumatology. Early age of presentation and familial tendencies are clues to the possibility of an underlying monogenic pattern. It is already known that autoimmunities are more common in relatives of children with SLE (3). Repetitive patterns in family members of lupus patients are in favor of underlying genetic causes. Mikhail M. Kostik et al. reported a case of 2 monozygotic twin brothers with simultaneous childhood SLE. This compelling case report emphasizes the possibility of monogenic origin in childhood SLE and justifies the need to perform WGS to study the spectrum of genetic variants associated with lupus.

Kwong et al. described a case of multiple autoimmune syndromes (MAS) comprising of type 1 diabetes, Hashimoto thyroiditis, and childhood-onset systemic lupus erythematosus (SLE). Although this is not a usual combination we expect to observe in polyautoimmunities, when repeated and reported, could help us integrate these new data into a more detailed understanding of disease pathogenesis and genetics, as well as possible interactions with environmental factors.

In the past decade the scientific community has drawn more attention to A20 Haploinsufficiency which is a rare autoinflammatory disorder with Behçet disease (BD) like characteristics caused by loss-of-function mutations in TNFAIP3 gene. Some of the most common symptoms of the disease are recurrent mucosal ulcers, periodic fever, musculoskeletal symptoms, skin lesions, and recurrent infections (4, 5). Aslani et al. reported two patients with A20 haploinsufficiency and HLH (6). Zanatta et al. discussed a patient with novel heterozygous mutation in TNFAIP3 who developed intestinal BD. Considering that there are not enough published cases on this topic, reports on this matter should be encouraged to reach better understanding about symptoms and complications.

Mauro et al. described the first case of Bardet–Biedl syndrome (BBS) associated with recurrent pericarditis (RP). Congenital heart diseases are the most common cardiac finding in BBS and acquired disorders like pericarditis are usually not expected in these patients. what is even more interesting, is the excellent response to treatment with anakinra in this reported patient. Contextually, Tsyklauri et al. suggested that patients with BBS have a higher prevalence of autoimmune disorders (7) but there is no published data to support the correlation between BBS and rheumatological or autoinflammatory disorders. This association is particularly compelling as it shows a new possible feature associated with BBS and suggests a plausible unknown underlying autoinflammatory mechanism in BBS.

Li et al. described a *de novo* missense variant in the KMT2D gene in a boy with distinctive facial features consistent with Kabuki syndrome (KS) and pulmonary hemorrhage who was diagnosed with Goodpasture's syndrome. To our knowledge, no other similar case of KS with pneumorrhagia is described in the literature. Although we cannot be certain whether Goodpasture's syndrome is part of the KS presentation or if it occurred coincidentally, this case report does expand on the phenotype of KS and the possible associated autoimmune disorders.

Lunz Macedo et al. described a patient with homozygous Factor H (FH) Deficiency who started manifesting signs of childhood-onset SLE at age 15 while his primary immunodeficiency was diagnosed at 5. It's well worth the mention that Case reports that relate autoimmune diseases like lupus to the complement system are often related to the defects in the classical complement pathway (8). However, the association between SLE and deficiencies in the components of the alternative pathway is uncommon. There is no previously published case of a patient with an initial presentation of FH deficiency with normal components of the classical pathway, who develops childhood SLE later in life. What makes this report even more noteworthy is the experimental treatment with Curcumin. Falcão DA and her team previously described a patient with FH deficiency in 2008 who showed a promising response to *in vitro* treatment with curcumin resulting in increased secretion of FH from the endoplasmic reticulum of the patient's fibroblasts (9). However, *in vivo* treatment with CURCUMIN derivatives “Theracurmin”, which was experimented with in this paper, did not result in an increase in the plasma levels of FH, C3, and FB in their patient and no change in the clinical and laboratory SLE parameters were observed.

Another interesting twist on Lupus is discussed by Li et al. which describes a case of SLE complicated with Lupus Podocytopathy (LP) and antiphospholipid syndrome (APS). We expect immune complex depositions in the mesangium, subepithelial or subendothelial regions in lupus nephropathy (10). However, Lupus podocytopathy is a non-immune complex-mediated type of lupus nephropathy. It is a newly described entity of non-immune complex-mediated lupus nephropathy and is not yet included in the updated 2018 International Society of Nephrology/Renal Pathology Society (ISN/RPS) classification of LN (11). There are very few reported pediatric cases of LP in the literature. but the coexistence of APS and LP in the same patient with SLE has not ever been reported in children. Li et al. study reinforces the need to consider the potential co-occurrence of APS and LP.

The increasing knowledge of inflammation and immunological pathways helps with new therapeutic options for rheumatologic disorders. These advances help in further understanding disease pathophysiology and progression and its associated complications, which support the stratification of patients to treatment pathways. The introduction of biological drugs has revolutionized the management of pediatric rheumatologic diseases, primarily in juvenile idiopathic arthritis (JIA), and has led to dramatic changes in the treatment strategies.

In the observational study performed by Xu et al. the efficacy and safety of Etanercept biosimilar recombinant human TNF-α receptor II: IgG Fc fusion protein (rhTNFR-Fc) is evaluated in 60 Chinese children with JIA and entesithis related arthritis. Their study indicated that the combination of rhTNFR-Fc and methotrexate (MTX) significantly improved the symptoms and disease activity of children with JIA.

Frkovic et al. present a 16-year-old girl with psoriatic JIA and bilateral Lipoma arborescens (LA) of her knees. Her Diagnosis was confirmed when she was 13 and primarily received conservative medical treatment (MTX) and TNF inhibitor—adalimumab was added later as a step-up approach, Which resulted in an almost complete regression of LA. LA is a non-specific reactive response to chronic inflammation associated with the proliferation of synovial villi, causing intraarticular lesions. During the last decade, a growing number of reports suggest that LA has an underlying inflammatory property. However, most clinicians are doubtful about the success of anti-inflammatory therapy in these patients and recommend synovectomy as the definitive treatment (12). Even though chronic synovial inflammation is the main pathogenesis of JIA, there are only several reports of LA in JIA patients. This case report is the first case of successful use of the TNF inhibitor adalimumab for treating bilateral knee LA in a patient with psoriatic JIA.

The use of biological treatments in MIS-C (Multisystem inflammatory syndrome in children) is also a point of focus in recent literature. MIS-C is a potentially life-threatening condition triggered by SARS-COV-2 infection. Since its first description, a huge effort has been made worldwide to better understand the pathogenesis and the clinical features of this novel entity to optimize therapeutical approaches (13).

La Torre et al. discussed a rare case of pulmonary vasculitis in an MIS-C patient who was not primarily responsive to IVIG and high-dose corticosteroids. Their use of sildenafil and high-dose anakinra as rescue therapy provided amazing results. It's intriguing to think that MISC-associated pulmonary vasculitis can benefit from biological treatments but more extensive studies are required to confirm these preliminary results.

Most MIS-C cases are managed with high dose corticosteroid therapy and immunomodulatory medications (14). However, the recent revisions of ACR recommendation agree that “*in mild cases, after evaluation by specialists with expertise in MIS-C, some patients may be managed with only close monitoring without immunomodulatory treatment*” (15). In the case series provided by Meneghel et al. the absence of laboratory and instrumental findings of cardiac involvement was the key point for a conservative approach, although in other cohorts in which a self-limited course has been reported cardiovascular dysfunction was described.

In conclusion, all these research efforts have significantly contributed to increasing the knowledge of pathophysiological, diagnostic, and therapeutic aspects of rheumatologic disorders.
